# Molecular docking and dynamic simulation analysis of BPTF with alkaloids

**DOI:** 10.6026/973206300220893

**Published:** 2026-02-28

**Authors:** Rohan Daniel Mammen, Jino Blessy

**Affiliations:** 1 Department of Bioinformatics, Sri Ramachandra Faculty of Engineering and Technology, Sri Ramachandra Institute of Higher Education and Research, Chennai, India

**Keywords:** Medulloblastoma, bromodomain PHD finger transcription factor (BPTF), docking, PyRx, desmond

## Abstract

Medulloblastoma, a malignant pediatric brain tumor, involves the bromodomain PHD finger transcription factor (BPTF) protein, an epigenetic regulator linked to tumor progression. Molecular docking and 100 ns molecular dynamics simulations were performed using PyRx and desmond to evaluate five plant based alkaloids Sanguinarine chloride, Coptisine, Chelerythrine, Nitidine and Chelidonine against BPTF. Coptisine showed high docking affinity of -6.8 kcal/mol, followed closely by Sanguinarine chloride and Chelerythrine (-6.6 kcal/mol). Sanguinarine chloride showed low toxicity in protox-III and high stability during simulations with consistent RMSD values (1.0-1.5 Å), indicating strong binding. Thus, Sanguinarine chloride emerged as the most promising BPTF inhibitor for potential anti-medulloblastoma therapy.

## Background:

Medulloblastoma is a highly malignant embryonal tumor of the central nervous system, occurring mainly in children under 16 years and rarely in adults. It is classified as a WHO grade IV tumor, with classical and desmoplastic subtypes being the most common. Histologically, medulloblastomas show high cellularity, small cells with a high nuclear-to-cytoplasmic ratio and coarse chromatin [1]. Tumors are staged using the Modified Chang Staging System, based on tumor size (T1-T4) and metastatic status (M) [2]. Clinical diagnosis of medulloblastoma remains challenging, often leading to delayed or incorrect diagnosis with serious consequences such as vision loss and brain herniation. Early symptoms may mimic gastrointestinal disorders, occasionally resulting in unnecessary appendectomy, or meningitis, where lumbar puncture can be harmful [3]. Standard medulloblastoma treatment involves surgery, craniospinal irradiation and chemotherapy, but recurrence is common due to heterogeneity and drug resistance [4]. BPTF, a chromatin-remodeling protein (17q24.3), regulates transcription via H3K4me2/3 and acetylated H4, promotes MYC-dependent proliferation and, when targeted, suppresses MAPK/PI3K-AKT, induces apoptosis and inhibits P-glycoprotein [5].

High-affinity targeting of BPTF inhibits growth and induces apoptosis in multiple cancer cell lines, highlighting its therapeutic potential [6]. As conventional therapies fail due to multidrug resistance, plant-derived alkaloids like nitidine and sanguinarine chloride, with antioxidant, anti-inflammatory, immunomodulatory and anticancer properties, show cytotoxic and EMT reversing effects, making them promising anticancer candidates [7]. This study evaluates five plant-derived alkaloids with established anticancer activity: sanguinarine chloride, nitidine chloride, coptisine, chelidonine and chelerythrine. These compounds induce apoptosis and inhibit cancer cell proliferation through modulation of key oncogenic pathways, including STAT3, PI3K/AKT/mTOR and redox-regulated survival mechanisms [8]. Molecular docking of the alkaloids with BPTF was performed using PyRx [9], followed by 100 ns molecular dynamics simulations using Schrödinger to assess binding stability [10]. Toxicity profiling was conducted using Protox-3.0 [11]. Therefore, it is of interest to identify BPTF-targeting plant alkaloids as potential therapeutic candidates for Medulloblastoma.

## Materials and Methods:

## Molecular docking:

The BPTF crystalline structure (PDB ID: 7K6R) was retrieved from Protein Data Bank [12]. The structure was obtained using X-ray diffraction at a resolution of 1.60 Å with an R-free value of 0.197. It is expressed in Escherichia coli. Cleaning of the protein was done using UCSF Chimera [13]. Non-standard residues along with water molecules were removed to simplify the structure. The binding sites of the protein were identified from PDBsum [14] (Laskowski 2022) for subsequent molecular docking studies. The binding residues were found to be Trp2950, Pro2951, Pro2955, Val2956, Asp2960, Ala2961, Tyr2964, Tyr2964, Asn3007, Phe3013. The 3D SDF structures of the plant-based alkaloids were obtained from the PubChem database [15]. Ligand preparation, including conversion of files to PDBQT format and performing energy minimization was done using PyRx. Molecular docking was performed using the AutoDock Vina engine within PyRx, with the active sites selected in Vina Wizard to define the docking region. The obtained protein-ligand complexes were visualized using UCSF Chimera to generate the combined structures and Discovery Studio Visualizer [16] was used to analyze hydrophobic interactions and hydrogen bonding.

## Molecular dynamics simulations:

Molecular dynamics (MD) simulations were performed for the BPTF-Alkaloid complexes using Desmond v7.6 [17], applying the OPLS_2005 force field for a 100 ns (nanosecond) simulation period. Each complex was solvated in an orthorhombic box (100 x 100 x 100 Å) with the TIP3P water model and periodic boundary conditions were applied. To neutralize the system, counter ions (Na^+^ and Cl^-^) were added. System equilibration was carried out using Desmond's standard protocol, which involves restrained minimizations and short MD simulations to allow gradual relaxation of the protein backbone and prevent structural distortions. The equilibrated systems underwent MD simulations in NPT ensemble conditions at 300 K and 1.01325 bars for 100 ns. Long-range electrostatic interactions were calculated using the particle-mesh Ewald (PME) method and a 9 Å cutoff was applied for van der Waals interactions. Trajectory analyses were conducted to evaluate the structural stability and dynamic behaviour of the BPTF-Alkaloid complexes. Root mean square deviation (RMSD) was analyzed to assess system stability, whereas root mean square fluctuation (RMSF) values were computed to determine residue flexibility. Additionally, hydrogen bond occupancy and persistence were monitored throughout the simulation to gain insights into the binding stability of the complexes.

## Toxicity prediction:

To evaluate the safety profiles of the selected alkaloids, toxicity prediction was performed using Protox-3.0. The SMILES structures of all compounds were submitted to the server to predict toxicity levels and all available endpoints were chosen to assess a range of toxicity endpoints which include hepatotoxicity, neurotoxicity, immunotoxicity and carcinogenicity. The toxicity profile of the five chosen plant-based alkaloids were predicted and analyzed.

## Results and Discussion:

**Figure 1A-E:**
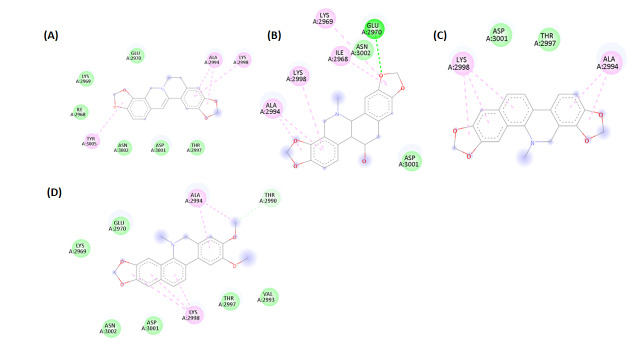
Molecular docking interaction diagrams of BPFT with (a) Chelerythrine, (b) Chelidonine, (c) Coptisine, (d) Nitidine, and (e) Sanguinarine chloride generated using BIOVIA Discovery Studio Visualizer.

Molecular docking was performed using PyRx software. The target protein, BPTF (PDB ID: 7K6R) was used for docking. The alkaloids Nitidine, Sanguinarine chloride, Coptisine, Chelidonine and Chelerythrine were docked against the target protein. Among all the tested compounds, Coptisine proved to have the strongest binding affinity of -6.8 kcal/mol followed by Chelerythrine and Sanguinarine chloride each showing binding affinities of -6.6 kcal/mol. Nitidine and Chelidonine each showed slightly weaker binding scores of -6.1 kcal/mol. As shown in Table 1, stronger binding affinities indicate stronger binding interactions and potential inhibition of BPTF for effective anti-cancer therapy. The binding interactions between BPTF and the alkaloids were analyzed using BIOVIA Discovery Studio Visualizer, which revealed the presence of hydrogen bonding, hydrophobic contacts and van der Waals interactions (Figure 1A-E). The toxicity profiles of the selected alkaloids were predicted using Protox-3.0. The toxicity levels were divided into 6 classes based on their median lethal dose LD50: class 1 (≤5 mg/kg): fatal if swallowed, class 2 (5-50 mg/kg): fatal if swallowed, class 3 (50-300 mg/kg): toxic if swallowed, class 4 (300-2000 mg/kg): harmful if swallowed, class 5 (2000-5000 mg/kg) may be harmful if swallowed and class 6 (>5000): non-toxic. Sanguinarine chloride and Nitidine exhibited toxicity class 4 with predicted LD50 of 778 mg/kg and LD50 of 1000 mg/kg respectively. Chelidonine exhibited similar class 4 toxicity with predicted LD50 of 460 mg/kg. Coptisine exhibited slightly higher toxicity of class 3 with predicted LD50 of 200mg/kg. Chelerythrine similar to the other compounds exhibited class 4 toxicity with predicted LD50 of 778 mg/kg (Table 2). Overall the chosen alkaloids were found to have minimal toxicity levels, suggesting their potential safety for further studies.

**Figure 2:**
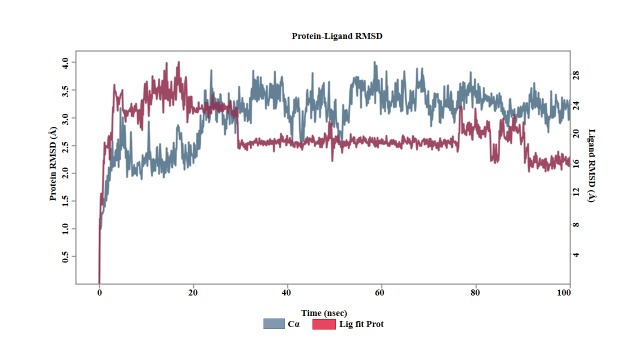
RMSD profile for the BPFT-Sanguinarine chloride complex.

**Figure 3:**
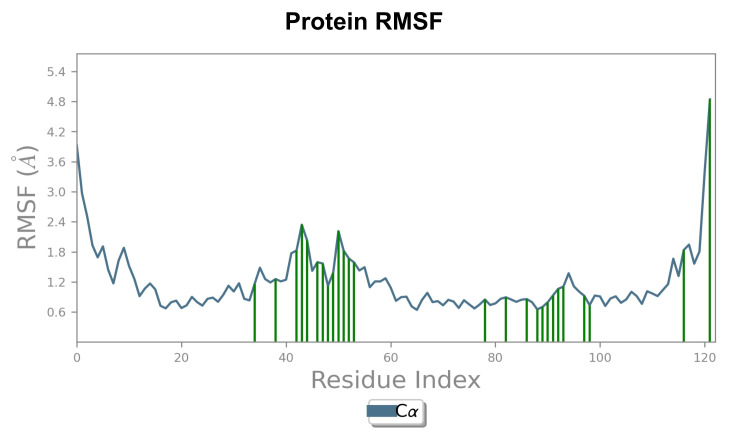
RMSF profile of the BPTF-Sanguinarine chloride complex.

Molecular dynamics simulation was done to analyze the internal motions of the receptor-ligand complex over time under flexible solvent conditions. To validate the binding of the complexes, simulations were performed using Desmond software. Throughout the 100 ns molecular dynamics simulations, all BPTF-ligand complexes showed overall protein stability, with Cα RMSD values falling between 2.4-3.3 Å. Differences were observed in ligand RMSD, displaying the stability of each compound within the binding pocket. Sanguinarine chloride demonstrated the highest stability, maintaining a ligand RMSD of 1.0-1.5 Å throughout the simulation, indicating strong and stable interactions with BPTF. Coptisine displayed steady stability with protein RMSD values fluctuating between 1.4-2.8 Å and ligand RMSD around 1.6-2.0 Å, signifying stable binding with moderate fluctuations. Chelerythrine showed moderate stability with fluctuations between 1.8-2.3 Å, while Nitidine and Chelidonine had greater variability (up to 5.0 Å and 4.5 Å, respectively), suggesting weaker or more transient binding. Overall, Sanguinarine chloride exhibited the most stable protein-ligand complex, highlighting its potential as the most promising inhibitor of BPTF among the compounds tested. The RMSD profile for the BPFT- Sanguinarine chloride complex is shown in Figure 2. The Root Mean Square Fluctuation (RMSF) analysis of the BPTF-ligand complexes highlighted variations in residue flexibility for the five alkaloids. The Chelidonine complex showed the highest RMSF fluctuations, ranging from 0.8 to 6.2 Å around residues 45-60 and suggesting higher flexibility. The Sanguinarine chloride complex exhibited RMSF values between 0.6 and 5.4 Å, with peaks around residues 40-55 showing a relatively stable protein-ligand interaction. Coptisine displayed RMSF fluctuations between 0.6 and 3.8 Å, with small peaks near residues 50-70, indicating stable binding and less residue mobility. Chelerythrine showed fluctuations between 0.6 to 4.8 Å, indicating consistent structural stability throughout the simulation, while Nitidine had RMSF values of 0.5 to 4.5 Å with peaks at residues 40-60 and 120. Overall, the RMSF values indicate that Sanguinarine chloride maintains better stability of the BPTF protein residues compared to the other alkaloids Figure 3. This study explored the ant-cancer potential of five plant-based alkaloids Nitidine, Sanguinarine chloride, Coptisine, Chelidonine and Chelerythrine against BPTF through molecular docking and molecular dynamics (MD) simulations.

**Figure 4:**
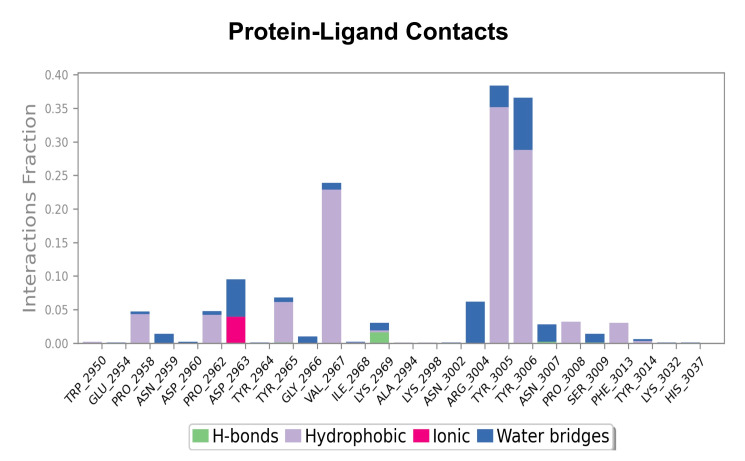
Protein-Ligand contacts of BPTF-Sanguinarine chloride complex.

All compounds displayed favorable binding affinities, with Coptisine showing the strongest docking score (-6.8 kcal/mol), followed by Sanguinarine chloride and Chelerythrine (-6.6 kcal/mol each). Though Coptisine had a slightly higher docking score, Sanguinarine chloride demonstrated better stability and binding consistency, making it the most promising inhibitor. Studies have shown that it induces ferroptosis through regulation of the ROS/BACH1/HMOX1 signaling pathway in prostate cancer, demonstrating its strong inhibitory potential in cancer therapy [18]. Interaction studies revealed that Sanguinarine chloride forms multiple hydrogen bonds, hydrophobic contacts and van der Waals interactions within the active site of BPTF, contributing to its stable complex formation Figure 4. MD simulations of 100 ns further supported these findings, showing that the BPTF-Sanguinarine chloride complex had the most stable RMSD values (1.0-1.5 Å) and minimal RMSF fluctuations, indicating strong interactions. Nitidine and Chelidonine showed higher fluctuations, indicating weaker binding. These results are consistent with earlier findings which reported that Coptisine and related alkaloids demonstrate notable antitumor activity in cells of gastric cancer, supporting the strong docking affinity and stability exhibited by Sanguinarine chloride in this study [19]. Toxicity predictions using Protox-3.0 classified Sanguinarine chloride as toxicity class 4 (LD_50_ = 778 mg/kg), reflecting moderate yet acceptable safety levels for pre-clinical evaluation. Though all tested alkaloids displayed inhibitory potential against BPTF, the results of this study identify Sanguinarine chloride as the most stable and promising candidate for a BPTF-targeted treatment for medulloblastoma.

## Conclusion:

We show that five plant-based alkaloids. Namely, Sanguinarine chloride, Coptisine, Chelerythrine, Nitidine and Chelidonine have optimal binding features with the BPTF protein, a key epigenetic regulator in medulloblastoma. All compounds showed strong binding affinities and stable interactions within the active site. Sanguinarine chloride displayed the highest stability and consistent binding during molecular dynamics simulations. Coptisine and Chelerythrine also showed favorable docking energies and stable complexes, while Nitidine and Chelidonine displayed moderate interactions. Thus, Sanguinarine chloride may be the promising lead for BPTF-targeted anti-medulloblastoma candidates.

## We thank MCC-MRF IP:

[Centre for Computational Informatics] for the Desmond molecular dynamics simulation.

## Disclosure statement:

No potential conflict of interest was reported by the author(s).

## Funding:

No funding was received.

## Ethical approval:

Not applicable.

## Informed consent:

Not applicable.

## Figures and Tables

**Table 1:** Binding affinities of alkaloids docked with BPTF

**Name of Compound**	**Binding Affinity (kcal/mol)**
Coptisine	-6.8
Chelerythrine	-6.6
Sanguinarine chloride	-6.6
Nitidine	-6.1
Chelidonine	-6.1

**Table 2:** Toxicity prediction results using Protox -3.0

**Compound**	**LD50 (mg/kg)**	**Toxicity class**	**Predicted Toxic Effects**
Sanguinarine chloride	778	4	Neurotoxocity
Nitidine	1000	4	Immunotoxicity
Chelidonine	460	4	Immunotoxicity
Chelerythrine	778	4	Immunotoxicity
Coptisine	200	3	Immunotoxicity
